# Lacto-Bacillin in Pernicious Anæmia

**Published:** 1909-02-20

**Authors:** 


### LACTO-BACILLIN IN PERNICIOUS ANÆMIA.

It is always very difficult to be sure whether the temporary recovery of any given case of pernicious anæmia is due to the drugs employed or not, but general experience points clearly to the fact that arsenic is the routine medicine for the disease, given in increasing quantities provided gastro-intestinal symptoms do not prohibit its use. So many cases of pernicious anæmia are prone either to vomiting, to diarrhœa, or to both, however, that alternative medicinal treatment is always welcome. One such alternative would seem to be lacto-bacillin; Dr. Gordon Dill showed a case of recovery from pernicious anæmia under its use before the Clinical Section of the Royal Society of Medicine recently.

The patient was a man of 34 years of age who had first suffered severely from diarrhœa in South Africa in 1901. He recovered, and apparently remained well until 1905, working as a plasterer and house painter. In July 1905 he was suddenly seized with severe abdominal pain and diarrhœa, followed a few days later by vomiting; both vomiting and diarrhœa persisted with considerable severity for nearly two months. From that time onwards he was constantly subject to vomiting and diarrhœa, the motions averaging eight a day. Dyspnœa and palpitations gradually ensued, and finally on October 10, 1907, admission to hospital became necessary. The blood-count on October 12 showed:—

| | |
|---|---|
| Red blood corpuscles ... | 1,468,000 per cub. mm. |
| Hæmoglobin ... ... | 32 per cent. |
| Colour index ... ... | 1.08 |
| Leucocytes ... ... | 3,500 per cub. mm. |
| Polymorphonuclears ... | 48 per cent. |
| Lymphocytes ... ... | 52 per cent. |

In films, some of the red cells exhibited punctate basophilia, and on subsequent examinations megaloblasts were noted more than once. From October 1907 to January 1908 the patient was treated with intestinal antiseptics and arsenic. Vaccines were also prepared from streptococci from his own bowel and from his own mouth, and were injected.

There was a little improvement, but not much, the blood-counts on January 23, 1908, showing:—

| | |
|---|---|
| Red blood corpuscles ... | 1,568,000 per cub. mm. |
| Hæmoglobin ... ... | 60 per cent. |
| Colour index ... ... | 1.9 |
| Leucocytes ... ... ... | 3,000 per cub. mm. |

Treatment by lacto-bacillin was begun on January 14, and continued until March 2.

After a month's interruption the treatment was recommended on April 3, when the count was:—

| | |
|---|---|
| Red blood corpuscles ... | 1,392,000 per cub. mm. |
| Colour index ... ... | 1.0 |

The condition got worse instead of better at first, so that by April 23 the blood showed:—

| | |
|---|---|
| Red blood corpuscles ... | 1,000,000 per cub. mm. |
| Colour index ... ... | 1.8 |

From the end of April onwards, however, steady improvement set in and was maintained, lacto-bacillin being continued until the end of September, except for an interruption in July. On September 30 the blood-count showed:—

| | |
|---|---|
| Red blood corpuscles ... | 4,800,000 per cub. mm. |
| Hæmoglobin ... ... | 100 per cent. |
| Colour index ... ... | 1.04 |
| Leucocytes ... ... ... | 6,400 |

It is, of course, impossible to assert that the excellent improvement thus recorded will be maintained. It is equally impossible to assert that the cure, even if temporary, is due to the lacto-bacillin. It is a sufficiently striking case, however, to warrant the trial of lacto-bacillin in future cases of pernicious anæmia, particularly if arsenic either fails to do good or produces symptoms which render its continued administration impossible.

